# Mechanical Properties and Applications of Recycled Polycarbonate Particle Material Extrusion-Based Additive Manufacturing

**DOI:** 10.3390/ma12101642

**Published:** 2019-05-20

**Authors:** Matthew J. Reich, Aubrey L. Woern, Nagendra G. Tanikella, Joshua M. Pearce

**Affiliations:** 1 Department of Material Science and Engineering, Michigan Technological University, Houghton, MI 49931, USA; mjreich@mtu.edu (M.J.R.); ngtanike@mtu.edu (N.G.T.); 2 Department of Mechanical Engineering–Engineering Mechanics, Michigan Technological University, Houghton, MI 49931, USA; alwoern@mtu.edu; 3 Department of Electrical and Computer Engineering, Michigan Technological University, Houghton, MI 49931, USA; 4 Department of Electronics and Nanoengineering, School of Electrical Engineering, Aalto University, 00076 Espoo, Finland

**Keywords:** 3D printing, additive manufacturing, distributed manufacturing, polymers, polycarbonate, recycling, waste plastic, extruder, upcycle, circular economy

## Abstract

Past work has shown that particle material extrusion (fused particle fabrication (FPF)/fused granular fabrication (FGF)) has the potential for increasing the use of recycled polymers in 3D printing. This study extends this potential to high-performance (high-mechanical-strength and heat-resistant) polymers using polycarbonate (PC). Recycled PC regrind of approximately 25 mm^2^ was 3D printed with an open-source Gigabot X and analyzed. A temperature and nozzle velocity matrix was used to find useful printing parameters, and a print test was used to maximize the output for a two-temperature stage extruder for PC. ASTM type 4 tensile test geometries as well as ASTM-approved compression tests were used to determine the mechanical properties of PC and were compared with filament printing and the bulk virgin material. The results showed the tensile strength of parts manufactured from the recycled PC particles (64.9 MPa) were comparable to that of the commercial filament printed on desktop (62.2 MPa) and large-format (66.3 MPa) 3D printers. Three case study applications were investigated: (i) using PC as a rapid molding technology for lower melting point thermoplastics, (ii) printed parts for high temperature applications, and (iii) printed parts for high-strength applications. The results show that recycled PC particle-based 3D printing can produce high-strength and heat-resistant products at low costs.

## 1. Introduction

The costs of additive manufacturing (AM) (notably material extrusion 3D printing) have dropped by several orders of magnitude with the open-source development of the self-replicating rapid prototyper (RepRap), which replaced proprietary fused deposition modeling (FDM) with the generic fused filament fabrication (FFF) [1,2,3]. With these cost declines came the real potential for a distributed manufacturing paradigm [4,5,6]: direct production by prosumers for significant cost savings compared to purchasing mass-manufactured products [7,8,9,10,11,12,13]. Economic analysis and the business literature support the growth of distributed manufacturing [14,15,16,17,18,19,20] because of the exponential rise of free 3D printable digital designs [12], which range from expensive scientific instrumentation [20,21,22,23,24,25,26,27] to everyday consumer items [10,11,12,13]. High return on investments (ROIs) are evident from the download substitution values using commercial 3D printing polymer filament [28,29]. Commercial filament, however, is generally marked up by more than five times to ten times the cost of raw plastic, which limits the cost savings and the concomitant deployment velocity of distributed additive manufacturing [30].

One method of overcoming the artificial cost barrier to distributed AM using material extrusion created by filament markups is to upcycle plastic waste into 3D printing filament with an open-source waste plastic extruder (recyclebot) [31]). The recyclebot process decreases the embodied energy of filament by 90% compared to traditional filament manufacturing [32,33,34,35]. This distributed recycling is a good example of an efficient circular economy [35], as it eliminates nearly all transportation embodied energy and pollution. Many recyclebot versions have been developed [36], including one that is primarily 3D printable and open-source [37]. Many types of waste polymers have been recycled into 3D printing filaments, including the most popular AM materials polylactic acid (PLA) [37,38,39,40,41] and acrylonitrile butadiene styrene (ABS) [35,42,43], as well as high-density polyethylene (HDPE) [31,43,44,45], polypropylene (PP) [45], polystyrene (PS) [45], polyethylene terephthalate (PET) [46], linear low-density polyethylene (LLDPE) and low-density polyethylene (LDPE) [47], elastomers [9], as well as composites using carbon reinforced plastic [48] and waste wood [49]-impregnated plastic. As the use of a recyclebot system introduces a melt extrude cycle that degrades the mechanical properties [50,51], recycling is limited to about five cycles [38,39] without blending with virgin materials. It is possible, however, to eliminate the need for filament entirely and print directly from particles, pellets, flakes, regrind, or shreds of recycled plastic. Such particle material extrusion (PME) 3D printers are also referred to as fused particle fabrication (FPF) or fused granular fabrication (FGF), and are becoming established in the academic [52,53,54], hobbyist [55,56], and commercial [57,58,59,60,61] arenas. Most systems were tested only with virgin pellets; however, two recent studies have shown enormous promise for PME printing of recycled materials in terms of both mechanical properties [62] and distributed manufacturing economics [63].

This study extends this potential to high-performance (high-mechanical-strength and heat-resistant) polymers using polycarbonate (PC) plastic. Recycled PC regrind was 3D printed with an open-source Gigabot X, and the material properties of the 3D-printed products were analyzed. A power and nozzle velocity matrix was used to optimize the print speed, and a print test was used to maximize the output for a two-temperature stage extruder for PC. ASTM type 4 tensile tests as well as ASTM compliant compression tests were used to determine the mechanical properties of PC and were compared with filament printing and the bulk virgin material. Three case study applications were investigated: (i) using PC as a rapid molding technology for lower melting point thermoplastics, (ii) printed parts for high-temperature applications, and (iii) printed parts for high-strength applications. The results are presented and discussed in the context of using waste PC directly for distributed manufacturing.

## 2. Materials and Methods

### 2.1. Materials

Clear PC regrind was provided by McDonnough Plastics, Fenton, MI, for $1/lb ($2.20/kg), to be used in the Gigabot X. The particle size characteristics of the starting material were quantified using digital imaging and the open-source Fiji/ImageJ [64] following a standard methodology [65] to determine the size distributions of the particles. Commercial PC filament with a consistent 2.85 mm diameter used for comparison printed on FFF was provided by Gizmodorks, Temple City, CA, USA [66].

### 2.2. PME 3D Printer and FFF 3D Printers

**Figure 1:**
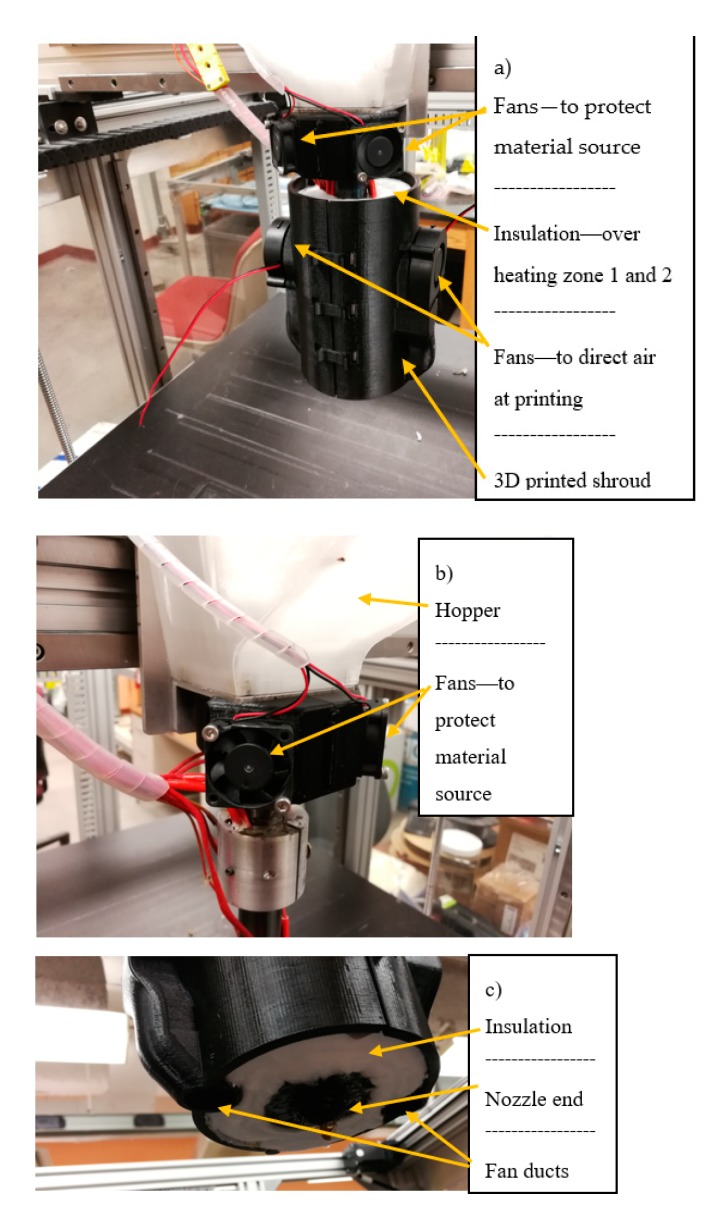
Gigabot X prototype additional cooling system showing (**a**) installation, (**b**) fan attachment at hopper, and (**c**) nozzle end.

A prototype Gigabot X (re:3D, Austin, TX, USA) [67,68] was used to print the recycled PC particles. 3D models were sliced with Slic3r [69], and the printer was controlled with Marlin Firmware [70]. Due to the high temperatures needed to print PC, and the lack of a print cooling system on the prototype Gigabot X, smaller prints (e.g., dimensions of only a few cm) were malformed. Unlike conventional FFF 3D printers, the extruder head on the Gigabot X is too large (250 mm long) to accommodate the two separate heating zones. The heating zones themselves are exposed and would melt any cooling fans attached near the extruder head. To overcome these challenges, 25 mm of fiber glass insulation was wrapped around the entire extruder system to contain the heat and allow for a cooling system to be attached. The cooling system itself was designed and released under an open-source license [71] and 3D printed out of PLA, which allowed two cooling fans to be attached (see Figure 1). The end of the extruder remained uninsulated and therefore still very warm, so the fans were designed to attach further up the extruder and two tunnels directed the air onto the printing surface. Comparisons between samples printed with and without the cooling system showed that perimeter walls were straighter and more uniform, and the cooling system was therefore used for the larger prints (tensile tests and applications).

To compare to conventional FFF, this study used both a desktop open-source Lulzbot TAZ 6 (Aleph Objects, Loveland, CO, USA) [72] as well as a conventional FFF-based Gigabot. Both printers were used to print PC filament with Lulzbot Cura 21.03 [73] slicer and Marlin firmware [69]. The TAZ 6 used a plexiglass enclosure to keep cool air drafts off the printer and keep some heat in. 

The printing parameters for all three printers are summarized in Table 1. Please note that the tensile bars were printed flat on the bed with the z axis oriented perpendicular to the print bed. The orientation of the 100% infill was set at 45 degrees, creating an x pattern down the length of the bars.

### 2.3. Temperature and Velocity Test

Testing procedures previously described for the PME platform [62] were used. The temperature vs. velocity matrix test for PC was accomplished by first identifying the upper and lower bounds of the printable temperature range. Typical desktop FFF-based PC printers print at around 250 to 310 °C, but to test a wide range of temperatures, the Gigabot X was set to continually extrude material, while the temperature was manually raised and lowered to find the physical upper and lower limits. An initial value of 260 °C, the melting temperature of polycarbonate, was slowly lowered until the PC solidified enough where extrusion was no longer possible for the stepper motor powering the screw. At 230 °C, the motor started skipping, so a lower limit was of 240 °C was used. On the opposite side of the temperature range, the Gigabot X was set to 300 °C, but due to moderate heat loss around the extruder, heating zone 2 only reached 270 °C and heating zone 2 reached 290 °C. These values were used as the upper limits. The tip of the nozzle (heating zone 1) and inside the barrel (heating zone 2) were set to every combination of temperatures in the printable temperature range at a resolution of 10 °C. A series of double lines was then printed at each temperature combination at print speeds from 5 to 50 mm/s in 5 mm/s increments. The printed lines were then massed on a digital scale (±0.01 g), and the line masses were compared to the theoretical line mass. The objective of this test was to find both the optimum print speed and the temperature settings for the extruder (both heat zones). The optimization was determined for settings, with the lowest standard deviation in the line weight for a set of lines and the print speeds across all the temperature settings that resulted in the heaviest line masses.

Once the print temperatures that obtained a close-to-theoretical value were found for each zone, a single-walled cylinder, printed as a spiral vase, was used to determine the extrusion multiplier and actual extrusion width of the polycarbonate. The cylinder, measuring 5 cm high and 10 cm in diameter, was printed and massed. The measured mass was compared to the theoretical mass, which was calculated using the volume of the material that made up the cylinder walls (theoretical) and the specific density of PC (theoretical). Proportions between the measured and theoretical masses, and multiple cylinder trials, were used to find the appropriate slicer extrusion multiplier and therefore the ideal flow rate for the settings tested to use for PC. After this was accomplished and 3D printing was carried out with the correct flow rate, digital calipers (±0.005 mm) were used to find the printed wall thickness. Input into the slicing software (Slic3r) [69], this value would ensure print quality for the remaining tests as the lines would be the correct width. 

### 2.4. Tensile Testing

Tensile testing was completed for the recycled PC using ASTM D638 Type 4 standard tensile bar geometry, and this same geometry was used for the FFF processed PC as outlined in Table 1 [74]. For the recycled PC, the bars were printed at the print settings that provided a close match to the theoretical mass found in Section 2.3 with 100% infill with an infill pattern of 45 degrees with respect to the long axis of the tensile bars. The specimens were then pulled until failure using a 10,000 lb. load cell (Model LCF455). The strain data was captured using the crosshead extension on the Universal Testing Machine.

### 2.5. Compression Testing

Compression testing was done using the same load cell (Model LCF455). Three different samples were chosen for compression: (1) cylinders with diameter 15 mm and length 30 mm (printed vertically), (2) rectangular prisms with length of 28 mm and thickness and width of 14 mm (printed vertically), and (3) rectangular prisms with length of 28 mm and thickness and width of 14 mm (printed horizontally), in accordance with the ASTM D695 standards. The specimens were printed with 100% infill in a 45-degree pattern. The compression specimens did not break, and hence they were tested to an arbitrary level of deformation (10%), in accordance with the ASTM standards. 

## 3. Results

### 3.1. Materials Size Distribution

**Figure 2:**
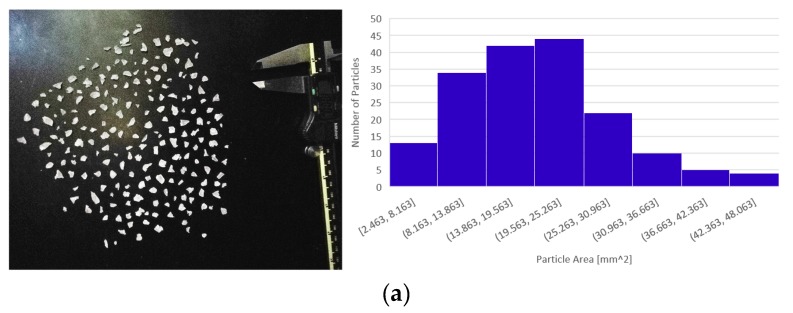

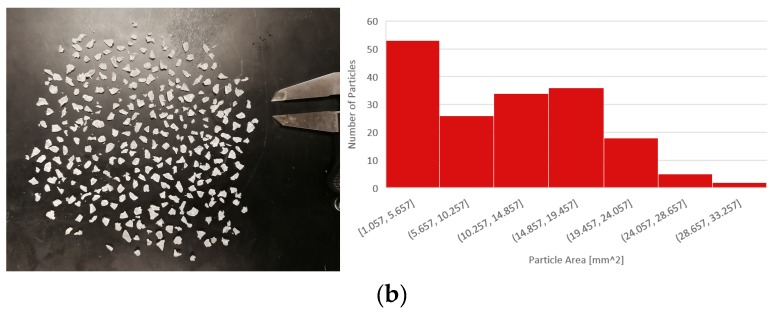
Recycled polycarbonate (PC) particles and size distribution, unsifted (**a**) and sifted (**b**).

Previous work [62] concluded that pellets with areas smaller than 22 mm^2^ could feed into the auger of the Gigabot X regardless of shape. A digital image of the input recycled PC is shown in Figure 2, which displays the size distribution of unsifted and sifted particle sizes, respectively. Initially, printing with the regrind particles without sifting was problematic due to the substantial PC particles size distribution. Larger particles would log themselves between the auger and the side of the hopper, causing the extruder to stop and the stepper motor to skip until the printer was shut down. To solve this problem, a sifter with holes approximately 5.5 mm in diameter was used to sift out the larger particles. As seen in Figure 2, the sizes of the sifted particles are considerably smaller than that of the unsifted particles, and mostly fall under the 22 mm^2^ recommendation. Without the larger PC particles, the auger was able to extrude the material without clogging and therefore produced a much more reliable print system. 

### 3.2. Printing Temperatures and Velocities

**Figure 3:**
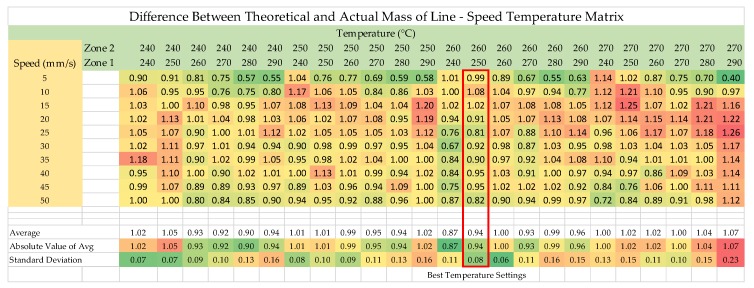
PC difference between the theoretical and actual mass of the line-speed temperature matrix.

The masses of the printed double lines were recorded in a matrix generated over the range of the operating temperatures for the two heating zones and the nozzle velocities from 5 to 50 mm/s. The difference between the theoretical and the actual mass of the line was recorded and is shown as a function of the nozzle speeds in Figure 3. The colors shown in Figure 3 are merely meant to act as eye guides and represent the relationship between the printed lines and theoretical mass; the darker green tone of the box around the number is, the closer to the theoretical value it is (the deeper red the further away). Figure 3 can be simply read by only looking at the numerical values. The ideal would be zero, i.e., the measured mass is equal to the theoretical. The average and standard deviation portion of the chart work similarly. The standard deviation measures how much each mass in a sample differs other masses in the same sample, and a lower standard deviation shows higher consistency in the print. 

Figure 3 shows the results for the recycled PC. The temperatures that provide values closest to the theoretical values were found to be 250 °C and 260 °C for heating zones one and two, respectively. As seen in Figure 3, none of the printed lines were very close to the theoretical mass. The best combination of temperatures was chosen based on its low standard deviation and respectably average value. Looking at data collected for 5 mm/s only, there is a positive correlation between temperature and proximity to the theoretical value. The higher the combined temperature of the heating zones, the closer the mass of the lines was to the theoretical values. Had this trend continued throughout all printing speeds, the highest temperature settings would have produced the best print, but due to the inconsistencies between the lines printed at faster speeds, this conclusion could not be met.

**Figure 4:**
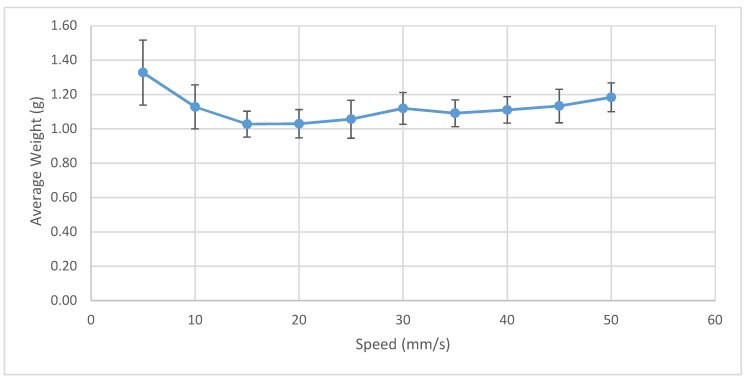
PC average mass as a function of the print speed.

Observing the trend in the average line weight speeds across the full temperature spectrum, line weight is maximized at print speeds of around 5 mm/s, as shown in Figure 4. At this print speed, the volumetric deposition rate is 5 mm^3^/s. In comparison, a traditional FFF printer at this speed, with a layer height of 0.2 mm and a 0.4 mm nozzle, will average 0.4 mm^3^/s. This translates to a 12.5 times increase in the 3D-printed part formation and therefore concludes that overall print time can be greatly reduced using the Gigabot X. 

### 3.3. Mechanical Testing

#### 3.3.1. Tensile Testing

The peak stresses/ultimate tensile strengths (UTS) for the specimens printed on the Gigabot X with PME/FGF ranged from 62.7 to 67.8 Mpa, with an average of 64.9 MPa and a standard deviation of 2.1 MPa. The peak stresses for the specimens on the FFF-based Gigabot were 63.0 MPa to 69.5 Mpa, with an average of 66.3 MPa and a standard deviation of 2.9 MPa. The peak stresses for the samples 3D printed on the FFF-based TAZ ranged from 59.5 to 66.8 Mpa, with an average of 62.2 MPa and a standard deviation of 2.8 MPa. The crosshead data shows a higher extension on the Gigabot X specimens (average 4.7 mm) when compared with the TAZ specimens (average 4.2 mm). The differences between the Gigabot X, FFF-based Gigabot, and the FFF-based TAZ samples could be due to several factors, including source of material, color of material, additives, layer height, temperature of extruder, speed of print, and size of nozzle. However, the samples are similar enough for the recycled material to be considered an alternative to the commercial filament on conventional FFF-based printers. However, it should be noted again that the Gigabot X is not appropriate for printing small samples with high resolution due to the large nozzle diameter.

#### 3.3.2. Compression Testing

The average compressive stresses at 10% deformation for the three sample geometries printed on the Gigabot X and the TAZ are shown in the Table 2. Compression tests show that components printed on the TAZ withstand higher stresses for the same deformation and have a lower standard deviation than the samples printed from recycled PC on the Gigabot X. 

For the Gigabot X, the rectangular prism sample printed horizontally has the lowest average stress and the highest standard deviation because the test specimens all were wider and longer at the base and tapered along the height. Thus, they sheared and broke into layers instead of compressing. Specimens printed vertically also had inconsitencies, which allowed compression of the smaller regions. However, this effect did not effect the type of deformation. Thus, the compression sample printed vertically has a higher strength. Furthermore, the prism sample has a higher strength than the cylindrical sample.

For the TAZ, there was little difference between the strength of the cylindrical sample and the prism sample printed vertically due to better reproduction at small scales with the smaller print head. The prism sample printed horizontally had higher strength than the prism sample printed vertically. It can be concluded that for applications involving small-scale products under compression, recycled PC printed with a Gigabot X cannot be used as a drop in replacement for PC on a conventional FFF printer. However, these effects would be expected to become less pronounced the larger the object printed. Future work is needed to quantify the effect of less consistent printing over the compressive strength of larger objects.

### 3.4. Case Studies

To determine the economics of printing PC on the Gigabot X, material and electric costs were analyzed. The material costs as a function of time were found by printing multiple standard parts at optimum speed and flow rate. Print times for each part were recorded; each part was massed on a digital scale (±0.01 g), and the average material flow for the was found to be 0.037 kg/hour. With a material cost of $2.20/kg, the Gigabot X would cost $0.074/hour to run based purely on materials. While printing at the temperatures, 260 °C for zone two, 250 °C for zone one, and 110 °C for the bed, and while the added cooling system was running, electricity use was monitored with a multimeter. With an average electricity cost of $0.1029/kWh in the US over all sectors [75], the Gigabot X costs approximately $0.13/hour in electricity to operate. Combined, the operational cost of the Gigabot X to print recycled PC is $0.204/hour.

**Figure 5:**
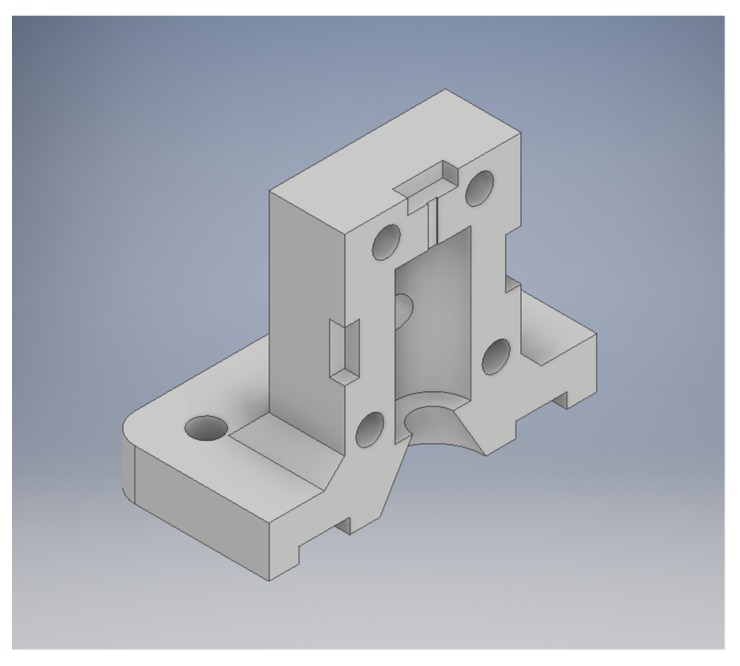
STL rendering of compression specimen mold.

Three case studies were used to test the recycled PC in PME/FGF applications. To take advantage of the high melting point of PC, low-cost molds were designed for use in a basic injection molding device. When many parts need to be made quickly, or when hard to print plastics are required, an injection molding machine and molds can be the best solution. This molding machine consists of a metal tube surrounded with heating elements and a plunger to push material through the hot zone into the mold. The tube is filled with plastic and the mold is then screwed onto the heated tube with a flange, and the plunger pushes the molten plastic into the mold cavity. Muhammad et al., using this method, 3D-printed molds from ABS after failed attempts at printing large water fixtures with recycled HDPE waste in the Solomon Islands [76,77]. Here, this method was improved by replacing ABS molds with molds from PC, which allowed for a larger quantity of parts to be made before the mold wore out as well as for higher temperature waste plastics to be used. An STL rendering of a mold made for compression specimens is shown in Figure 5. In addition, case studies of a consumer high-temperature application (steam cleaner) and high-strength application (ice scraper) were fabricated and tested.

#### 3.4.1. Rapid Molding Technology for Lower Melting Point Thermoplastics

Waste PC was successfully manufactured into a mold (design shown in Figure 5) that can be used for rapid molding of lower melting point thermoplastics. 

**Figure 6:**
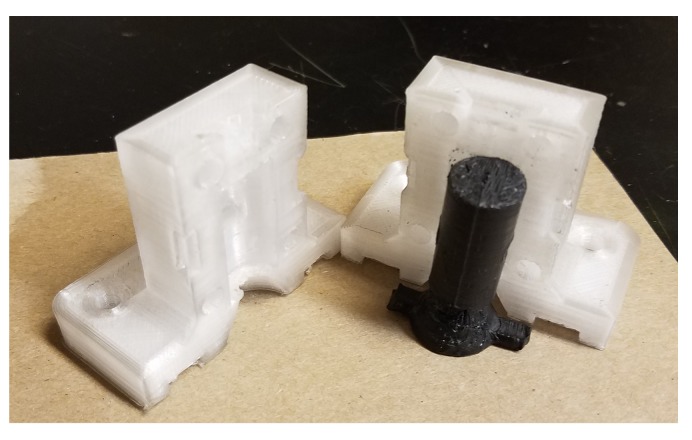
3D-printed compression specimen mold with molded part.

This is demonstrated in Figure 6 for a TPU composite. Following [76,77], this technique can be used for distributed recycling of waste polymers in resource-constrained communities. It can also be used in any setting that needs multiple copies of a plastic product and is particularly effective for hard-to-print thermoplastics, as well as in abrasive composites that may damage a recyclebot or a tool like the Gigabot X. 

#### 3.4.2. High-Temperature Applications

**Figure 7:**
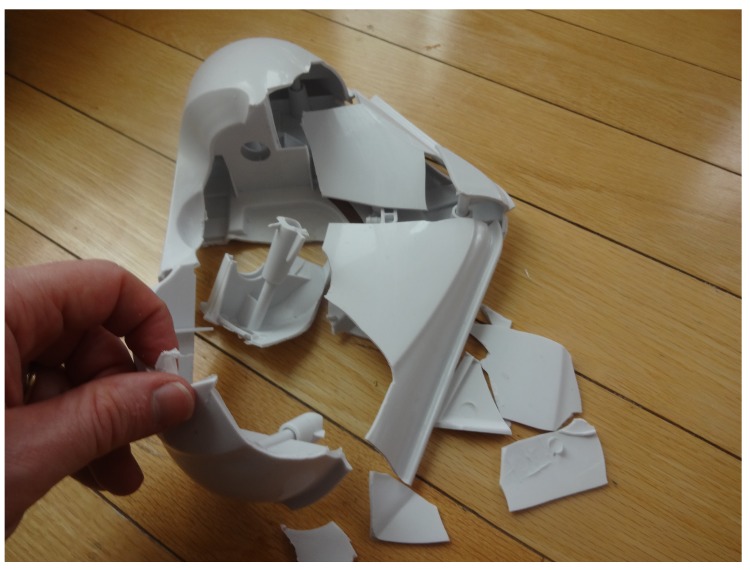
Broken commercial proprietary steamer head. The plastic became brittle and disintegrated under normal use.

**Figure 8:**
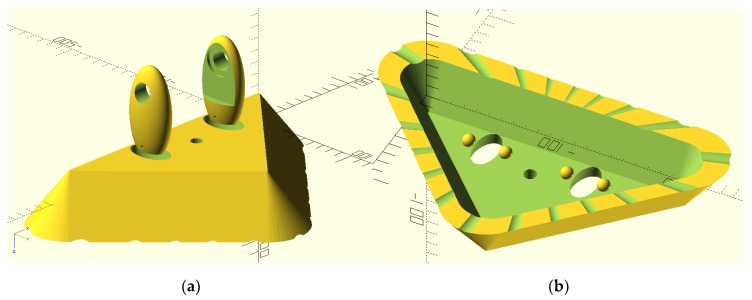
Rendering of OpenSCAD design of replacement steamer (**a**) top view and (**b**) bottom view.

It has long been the practice of some consumer product vendors to manufacture for obsolescence as well as use lower-cost non-optimal materials. An example of this is shown in Figure 7, a home floor steamer whose outer plastic has disintegrated under normal use. There is no replacement part available on the market, so a replacement was designed to be optimized for ease in 3D printing (as shown in Figure 8) and made available under an open-source license [71]. The design is made specifically to be 3D printed and uses no connecting hardware (as was done in the commercial design). The horizontal holes lock the three components together using the common filament rivet technique. Filament of PC can be produced from PC shards with the Gigabot X by simply extruding into air. The final assembly is completed using a soldering iron to melt the rivets into place. The only design feature used from the original design for the replacement was basic geometry to fit the existing cloth mop head, the sizing of the holes to fit onto the mop handle, and the hole for the steam tube. Thus, the new design is a true new design, not a reverse-engineered one or a copy.

**Figure 9:**
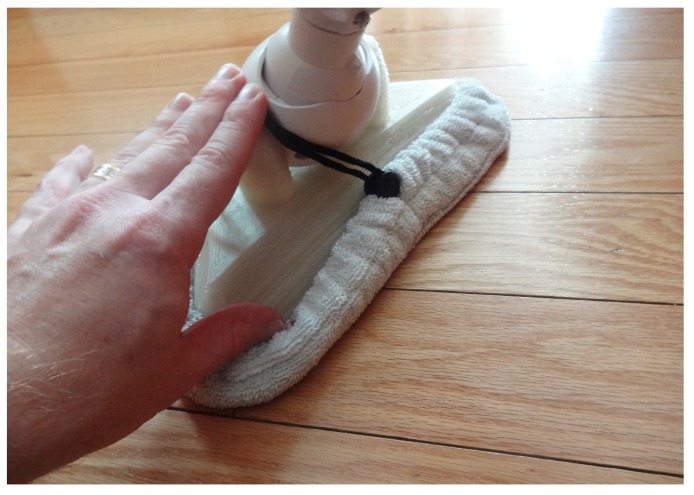
Waste PC 3D-printed steamer head shown assembled on steamer.

The steamer head as shown in Figure 9 was 3D printed in PC waste and tested. It successfully performed the same function—essentially guiding the steam from a flexible nozzle to the floor where a cloth was used for wiping (note: cloth being held back in Figure 9 to see 3D-printed part). This 3D print, which used about 520 g of waste PC and cost about US$2.50 to print successfully (including material and electricity costs), eliminated the need to buy an entirely new replacement steamer, which can be purchased from US$59–US$199 on Amazon [78]. Thus, printing replacement parts with recycled PC on the Gigabot X represents a 95% to 99% savings from purchasing new consumer goods made from materials with designed obsolescence.

#### 3.4.3. Printed Parts for High-Strength Applications

**Figure 10:**
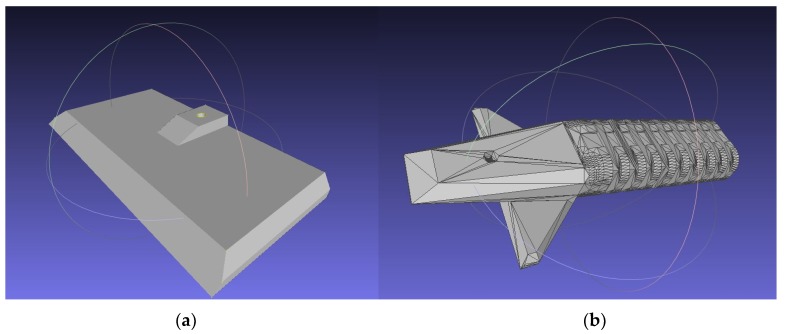
Meshlab rendering of STLs for (**a**) blade and (**b**) handle of snow scraper.

**Figure 11:**
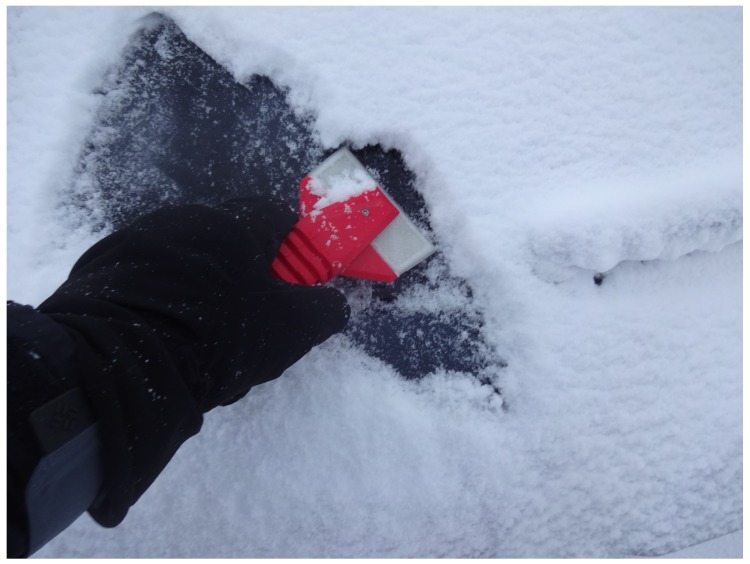
3D-printed ice/snow scraper in use. The blade is recycled PC and the handle is polyactic acid (PLA).

To exhibit the high-strength properties of the PC, an open-source (CC-BY licensed) car window ice scraper designed in Russia with interchangeable blades [79] (Figure 10) was printed and tested (Figure 11). The blade (Figure 10a) was printed in recycled PC. The handle was printed out of poly lactic acid (PLA) (Figure 10b) on a TAZ 6. 

The strength of the PC gives the blade of the scraper the desired ability to scrape away ice and snow buildup on windshields without breaking or chipping. The recycled PC blade was tested over a winter of normal use in the upper peninsula of Houghton Michigan, which receives an average of 5.54 m of snowfall during the roughly 6-month winter. The blade remained strong and functional. In addition, being able to bend without breaking because of fabrication with PC enabled the blade to form to the windshield while in use.

## 4. Discussion

Previous research has shown that 3D printing recycled particles with a Gigabot X as opposed to commercially sold filament can substantially lower the costs of printing, decrease print time (because of the large nozzle), and increase ease of printing large objects [62,63]. This latter point is particularly important when printing objects that would use more than one spool of filament [80]. These conclusions were confirmed here with PC, as the costs for printing recycled PC material are roughly 2.5% of the costs of printing with commercial PC filament. The case studies showed that distributed manufacturing of recycled PC with an PME/FPF printer was useful over a wide range of applications that require both (e.g., molding) or either high temperature resistance (e.g., steamer head) or high strength (e.g., ice scraper). PME/FPF of recycled PC allows for a wider range of needs to be filled. The large nozzle size and increased volumetric deposition rate grants the Gigabot X the ability to manufacture big components able to resist heat, and some high-strength properties. Applications include sporting equipment, personal protection, household appliance fixes, scientific instrumentation, and hand tool modifications. There remain some challenges in some areas of the world. For example, although China has a recycling symbol [81] for polycarbonate (58) in the U.S., it is grouped with dozens of other polymers in the other category (7) [82]. The open-source 3D printer community has already discussed the adoption of a voluntary recycling code based on the more comprehensive Chinese system [83]. This may be of modest help now as distributed manufacturing is still in its infancy; however, as the global value chains [16] shift because of the superior economics of distributed recycling and manufacturing, the prosumer community will benefit from regulations that stipulate that manufacturers identify the materials in their products [84]. This can go beyond primary polymers to list colorants, plasticizers, and other additives to better improve the ease of recycling.

This study also showed that the tensile strength of 3D-printed parts made from recycled PC was comparable to those made with conventional filament used in a conventional RepRap-class FFF 3D printer. Prior work on other materials [85,86] has shown that RepRap-class FFF 3D-printed parts are often superior to the more constrained printing parameters used in proprietary fuse deposition modeling (FDM) machines. For small nozzle heads, using smaller type iv FFF tensile samples was shown to be adequate [87], and those results were also confirmed here with the larger nozzle size of the PME/FPF head used. However, the larger head was found to be a problem when attempting to obtain reliable small-scale compression samples. Further work is required for large-volume compression samples to determine large area compressive strength, as the results shown here can be considered a pessimistic under estimate of the recycled PC compressive strength. In addition, in the results shown by the double line tests, higher print temperatures increase the line mass, but only for 5 mm/s. Future work at higher temperatures for 5 mm/s and other speeds may lead to ideal temperature combinations and improve the mechanical properties of the PC further. Printing at higher temperatures, which is possible because of the improved insulation around the extruder, might increase layer adhesion and improve print quality as well. Full optimization studies can also be run on layer height, print speeds, temperature, print orientations, etc. These optimization studies would ideally compare FFF with the same nozzle size to the PME/FPF for both recycled and virgin PC. In addition, past work on other materials that have gone through multiple 3D printing and recycling cycles have shown a degradation in the polymers and a reduction in mechanical strength [38,39]. Similar studies should be performed on multiple cycles of PC on both a PME/FPF printer and a conventional FFF printer to quantify the benefits of the reduced numbers of melts necessary in PME per cycle. Finally, the entire PME/FPF apparatus could be enclosed, which would again be likely to improve printed part quality and is left for future work. 

Fortunately, there has been a noticeable increase in recent studies on 3D printers capable of using pellets as a feedstock, including those derived from metal injection molding [88,89], double stage screw extruders [90], RepRap technology [91], and industrial robots [92]. The study here provided further evidence that such machines could be used for recycled materials, as PC is a more challenging printing polymer because of its higher temperatures and higher strengths (e.g., if bed adhesion is sub-optimal and the print begins to deform, a PC print will often fail because of mechanical contact with the print head more often than weaker polymers like ABS). There is thus considerable future work to begin to apply PME/FPF printing to composites (ideally made from waste) such as for conductive materials [93] and flexible materials [94]. The results of both this study and previous studies on FGF and PME/FPF infer this type of 3D printing will play a larger role in the additive manufacturing industry in the future. 

## 5. Conclusions

The results of this study found that sifted recycled PC shards could be effectively used as feedstock for PME/FPF additive manufacturing directly with a Gigabot X. Printing parameters that obtained close to theoretical values of mass were found to be 250 and 260 °C for printing zones 1 and 2, respectively, at 5 mm/s. The average peak stress for recycled PC on the Gigabot X prints was 64.9 MPa and a standard deviation of 2.1 MPa, indicating that it can be used as a drop-in replacement for 3D-printed PC parts on a standard FFF printer using conventional filament. The compressive testing was less conclusive, although it is clear that inconsistent depositions on small scales create weaker compressive strength than the same material deposited with a smaller print head. Substantially faster printing speeds are possible (10X) because of a larger printing nozzle diameter without a substantial decrease in performance, although future work is needed to further optimize the system for recycled PC shards. Recycled PC particles has been proven to be an inexpensive and useful material to use for additive manufacturing on PME/FPF 3D printers. The viability of recycled PC particles offers a wide range of uses that can replace more expensive solutions for prosumers and industry alike. 

## Figures and Tables

**Table 1:** 3D printer settings for all additive manufacturing (AM) systems tested.

3D Printer	FFF TAZ	FFF Gigabot	PME/FPF Gigabot X
Layer height	0.3 mm	0.4 mm	0.5 mm
Nozzle diameter	0.5 mm	1.2 mm	1.75 mm
Number of outer shells on all surfaces	2 *		
Orientation of Infill	Rectilinear at 45 degrees with respect to long axis of tensile bars		
Infill	100%		
Speed	30 mm/s	20 mm/s	Variable, 10 mm/s
Temperature	270 °C	250 °C	250–260, zone 1 and 2 (after velocity temperature test)
Bed temperature	110 °C		

* Note the resultant shell thickness varies for the different AM systems because of different nozzle sizes.

**Table 2:** Compression tests.

Average Stress at 10% Deformation (MPa)				
Open-Source 3D Printer→ Sample Geometry↓	Gigabot X	Std Deviation	TAZ FFF	Std Deviation
Cylinder	50.8	5.2	68.1	1.4
Prism (vertical)	60.9	7.3	70.4	1.3
Prism (horizontal)	43.3	17.6	77.3	1.8
